# Identification of defensin-encoding genes of *Picea glauca*: characterization of PgD5, a conserved spruce defensin with strong antifungal activity

**DOI:** 10.1186/1471-2229-12-180

**Published:** 2012-10-05

**Authors:** Pere Picart, Anna Maria Pirttilä, Dora Raventos, Hans-Henrik Kristensen, Hans-Georg Sahl

**Affiliations:** 1 Institut für Mikrobiologie und Biotechnologie, der Universität Bonn, Meckenheimer, Allee, Bonn, 167, 53115, Germany; 2 Department of Biology, University of Oulu, Linnanmaa, Oulu, PO Box 3000 A6, FIN-90014, Finland; 3 Novozymes A/S, Krogshoejvej, Bagsvaerd, 36, DK-2880, Denmark

**Keywords:** Spruce defensins, Endophyte, Antifungal activity, Membrane permeabilization

## Abstract

**Background:**

Plant defensins represent a major innate immune protein superfamily that displays strong inhibitory effects on filamentous fungi. The total number of plant defensins in a conifer species is unknown since there are no sequenced conifer genomes published, however the genomes of several angiosperm species provide an insight on the diversity of plant defensins. Here we report the identification of five new defensin-encoding genes from the *Picea glauca* genome and the characterization of two of their gene products, named PgD5 and endopiceasin.

**Results:**

Screening of a *P. glauca* EST database with sequences of known plant defensins identified four genes with homology to the known *P. glauca* defensin *PgD1*, which were designated *PgD2-5*. Whereas in the mature PgD2-4 only 7–9 amino acids differed from PgD1, PgD5 had only 64% sequence identity. *PgD5* was amplified from *P. glauca* genomic DNA by PCR. It codes for a precursor of 77-amino acid that is fully conserved within the *Picea* genus and has similarity to plant defensins. Recombinant PgD5, produced in *Escherichia coli*, had a molecular mass of 5.721 kDa, as determined by mass spectrometry. The PgD5 peptide exhibited strong antifungal activity against several phytopathogens without any effect on the morphology of the treated fungal hyphae, but strongly inhibited hyphal elongation. A SYTOX uptake assay suggested that the inhibitory activity of PgD5 could be associated with altering the permeability of the fungal membranes. Another completely unrelated defensin gene was identified in the EST library and named *endopiceasin*. Its gene codes for a 6-cysteine peptide that shares high similarity with the fungal defensin plectasin.

**Conclusions:**

Screening of a *P. glauca* EST database resulted in the identification of five new defensin-encoding genes. *PgD5* codes for a plant defensin that displays non-morphogenic antifungal activity against the phytopathogens tested, probably by altering membrane permeability. PgD5 has potential for application in the plant biotechnology sector. *Endopiceasin* appears to derive from an endo- or epiphytic fungal strain rather than from the plant itself.

## Background

Plants are exposed to a diverse array of pathogens and pests and their survival depends on different mechanisms for self-defense. Such defenses include physical cell wall barriers [1], as well as the production of a diverse range of molecules which can inhibit the growth of microbial pathogens [2-6]. Among the latter, cationic antimicrobial peptides such as defensins, are a most relevant and large family of defense compounds [7-9].

Plant defensins are characterized as small globular, basic, cysteine-rich proteins (45–54 amino acids), containing a triple-stranded antiparallel β-sheet and one α-helix that are stabilized into a compact shape by four disulfide bridges [10-14]. These bridges form a cysteine-stabilized α-helix β-sheet motif (CSα/β) that is well conserved in peptides with antimicrobial activity. Two additional structural motifs have been described in the plant defensin structure, namely the α-core, encompassing the loop connecting the first β-strand and the α-helix, and the γ-core containing the hairpin loop connecting β-strands 2 and 3 (Lβ2β3) [15,16]. Despite the tertiary structure being strongly conserved in plant defensins, the similarity on the primary sequence level is limited to eight cysteine residues, two glycines, one aromatic residue and a glutamic acid in the defined positions [17]. Variations in the amino acid sequences are reflected by small changes in the spatial display of the loops that contribute to the wide range of biological activities observed in these peptides, as a single amino acid substitution can change the spectrum of activity.

Unlike the insect and mammalian defensins, which are mainly active against bacteria [2,8,18,19], plant defensins, with a few exceptions, do not exhibit antibacterial activity [20]. They inhibit the growth of a broad spectrum of fungal plant pathogens, such as *Fusarium oxysporum*, *Verticillium dahliae* and *Botrytis cinerea*, but also of *Saccharomyces cerevisiae* and human pathogenic fungi such as *Candida albicans* at very low concentrations *in vitro*[20]. The precise mode of action of plant defensins is still unclear, and for most plant defensins the molecular components involved in signaling and putative intracellular targets remain unknown [21,22]. Only for the defensin Rs-AFP2 from *Raphanus sativus* and the defensin Dm-AMP1 from *Dahlia merckii*, a putative target in the fungal membrane has been identified [23,24]. More recently, it was demonstrated that plant defensins can be internalized into the cytoplasm and interact with specific intracellular targets [25,26]. Moreover, some members of the plant defensins family were found to have additional activities *in vitro*. These include the inhibition of α-amylase [27-29], protein translation [30,31] and proteases [32], as well as zinc tolerance mediators in plant [33], ion channel blockers [34,35], enzymatic activity involved in ascorbic acid redox state [36,37] and activity towards mammalian cells [38-40].

Plant defensins and their antimicrobial effects have been reported from many angiosperm plants, including monocots and dicots. The genome of *Arabidopsis thaliana* contains 14 different defensin genes that respond to various stresses [8]. However, polypeptides with similar properties have not been well studied in gymnosperms. The availability of a substantial expressed sequence tag (over 300 000 ESTs) resource developed for *Picea glauca*[41,42] has resulted in the identification of only one expressed defensin gene, *PgD1*[43], and one antifungal protein [44]. This prompted us to perform a wide bioinformatics analysis to screen for novel spruce defensins.

Here, we describe the identification of new *Picea* defensin genes, and the characterization of one of the defensin peptides, PgD5. The identification of new defensins from *P. glauca* expands our knowledge on conifer genomics and raises interest to study the potential of spruce defensins as fungicidal agents.

## Results

### Defensin-like peptides in *Picea glauca* genome

**Figure 1:**
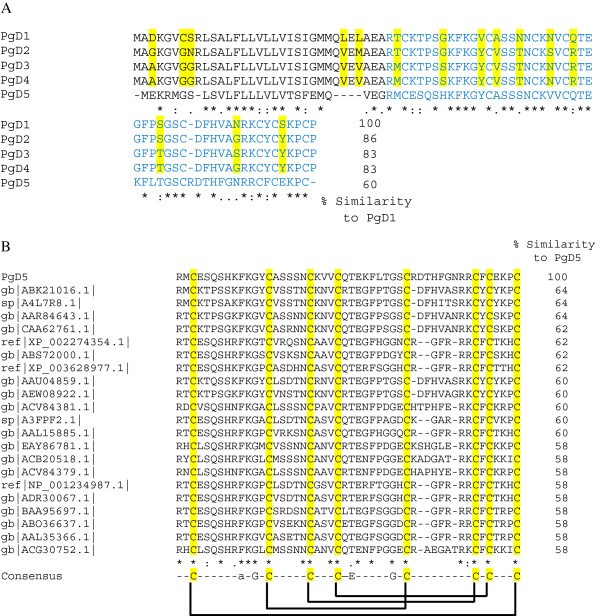
**Alignment analysis of the deduced amino acid sequences of *Picea glauca *defensin-encoding genes discovered by database searches.** (**A**) PgDs retrieved by screening the *P. glauca* EST database using the amino acid sequence of *P. glauca* defensin 1 (PgD1). The percentage similarity compared to PgD1 is indicated in the last column. The major differences between PgD1 and closely related PgD2, PgD3 and PgD4 are indicated by yellow. The deduced region of mature PgDs is indicated by blue amino acids. (**B**) Alignment analysis of the deduced mature region of PgD5 peptide with other members of the defensin family. The percentage similarity compared to PgD5 is indicated in the last column. [GenBank:ABK21016.1] unknown *Picea sitchensis*; [Swiss-Prot:A4L7R8.1] defensin 2 *Pinus sylvestris*; [GenBank:AAR84643.1] defensin PgD1 *Picea glauca*; [GenBank:CAA62761.1] putative gamma-thionin protein *Picea abies*; [GenBank:XP_002274353.1] Vv-Amp defensin *Vitis vinifera*; [GenBank:ABS72000.1] putative defensin 1 *Olea europaea*; [GenBank:XP_003628977.1] defensin *Medicago truncatula*; [GenBank:AAU04859.1] defensin precursor *Ginkgo biloba*; [GenBank:AEW08922.1] hypothetical protein *Pinus radiata*; [GenBank:ACV84381.1] defensin precursor *Triticum durum*; [Swiss-Prot:A3FPF2.1] defensin-like protein *Nelumbo nucifera*; [GenBank:AAL15885.1] putative gamma-thionin *Castanea sativa*; [GenBank:EAY86781.1] hypothetical protein *Oryza sativa*; [GenBank:ACB20518.1] defensin precursor *Saccharum officinarum*; [GenBank:ACV84379.1] defensin precursor *Triticum aestivum*; [GenBank:NP_001234987.1] protease inhibitor precursor *Glycine max*; [GenBank:ADR30067.1] defensin D2 *Phaseolus vulgaris*; [GenBank:BAA95697.1] thionin like protein *Nicotiana tabacum*; [GenBank:ABO36637.1] defensin protein *Solanum lycopersicum*; [GenBank:AAL35366.1] defensin protein precursor *Capiscum annuum*; [GenBank:ACG30752.1] defensin *Zea mays*. The consensus sequence containing the eight cysteine residues, two glycines, an aromatic residue and a glutamic acid, common to all plant defensins, is indicated below. The disulfide bridge organization within the PgD5 sequence is indicated below the consensus sequence.

The *P. glauca* EST database was first screened with the amino acid sequence of *Picea glauca* defensin 1 (PgD1, GenBank:AAR84643) by the TBLASTN program. TBLASTN is especially suitable for the discovery of distant homologues with a conserved sequence motif [45]. This initial screening yielded four different EST hits, GQ03918_C16, GQ02811_I12, GQ03707_G02 and GQ01307_A13, which shared similarity to *PgD1*. The complete coding sequences of the isolated ESTs were then named *P. glauca defensin 2* to *5* (*PgD2-5*), respectively. When the deduced coding sequences of the new *PgDs* were compared to PgD1, high similarity was observed for PgD2-4 with 83-86% sequence identity (Figure 1A). In contrast, only 60% identity was observed when PgD1 was compared with PgD5.

Alignment analysis of the deduced amino acid sequences of *PgD2-5* revealed that *PgD2* codes for a precursor of 83 amino acid with the highest similarity to defensin 3 from *Pinus sylvestris* [GenBank:JN980401] at the 93% similarity level (data not shown). *PgD3* and *PgD4* code for 83 amino acid peptides sharing 100% and 99% identities with an unknown protein from *Picea sitchensis* [GenBank:ABK21016], respectively (data not shown). The deduced amino acid sequences of *PgD3* and *PgD4* were identical, except for a substitution of Ser for Gly in position 73 (numbering according to PgD1 from *P. glauca*) (Figure 1A). *PgD5* codes for a precursor of 77-amino acids. Alignment of the deduced amino acid sequence revealed that the mature region of PgD5 shares high similarity with members of the gymnosperms, displaying 64% homology to both the Scots pine defensin 2 [Swiss-Prot:A4L7R8.1] [46] and the defensin 1 from *Picea glauca* [GenBank:CAA62761.1] [43], respectively, as well as to unrelated plant defensins from different families (Figure 1B). For instance, it displays 62% similarity to defensin vv-AMP1 from *Vitis vinifera*[47] and 62% similarity to putative defensin 1 from *Olea europaea*[48]. PgD5 shares 12 conserved amino acids, including eight cysteine and two glycine residues, as well as one glutamic acid and one aromatic residue at conserved positions. These amino acids are common to all plant defensins [17]. Disulphide-bridge analysis done with DIpro confirmed that the eight cysteine residues of PgD5 are connected by four disulfide bridges (Figure 1B).

When the *P. glauca* EST database was further screened with the amino acid sequences of several other gymnosperm defensins, no additional new defensins were identified. Surprisingly, screening of the *P. glauca* EST database with the amino acid sequence of plectasin [Swiss-Prot:Q53I06.1] [49] yielded one EST hit, GQ0132.B7_K03, which shared 58% similarity to plectasin ( Additional file 1). The complete coding sequence of EST GQ0132.B7_K03 was 273 bp in size and codes for a predicted 90-amino acid peptide. SignalP results showed that the first 20 amino acids code for a signal peptide followed by a 30-amino acid pro-peptide and a mature peptide of 40 amino acids ( Additional file 2). Comparative analysis of the deduced amino acid sequence of mature peptide with other members of the defensin family revealed that the peptide shares a disulphide bridge pattern common to defensins of fungi, insects and other invertebrates [50,51]. It contains three disulphide bridges in contrast to all plant defensins that typically have four disulphide bridges ( Additional file 3).

The most distantly related *PgD5* and the gene encoded within EST GQ0132.B7_K03 were selected for further analysis. In both cases, the cDNA library containing the corresponding genes was exclusively isolated from actively elongating roots tips free of mycorrhiza.

### Genomic and *in silico* characterization of *PgD5* and *endopiceasin*

**Figure 2:**
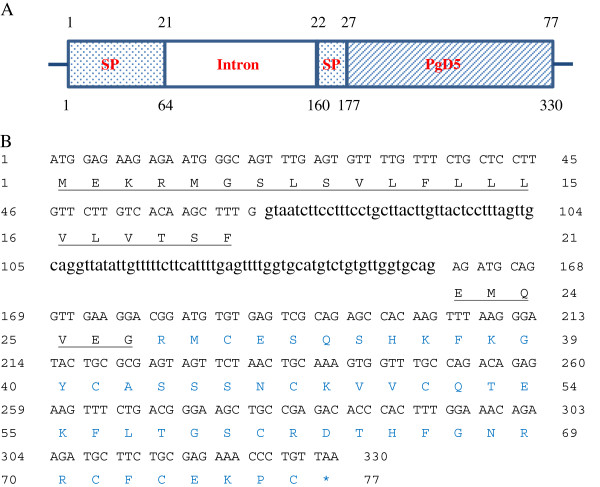
**The genomic structure of the *PgD5 *gene.** (**A**) Gene structure of *PgD5*. The numbering above the scheme refers to the amino acid sequence of the PgD5 precursor, and the figures below the scheme correspond to the nucleotide sequence of the *PgD5* gene. (**B**) The complete coding sequence and the deduced amino acid sequence of *PgD5* encoded within the EST GQ01307_A13. The underlined amino acids represent the signal peptide (1–27 aa) while blue amino acids indicate the mature peptide (28–77 aa). The intron (65–160 nts) is indicated by lowercase letter.

Primers were designed based on the EST GQ01307_A13 sequence and PCR was carried out to amplify the complete coding sequence of *PgD5* using genomic DNA of *P. glauca*. Genomic amplification of the sample resulted in a product of about 330 bp and a comparative analysis with the EST GQ01307_A13 revealed that a 96 bp-intron interrupts the predicted signal peptide (Figure 2A). SignalP analysis showed that the first 27 amino acids code for a signal peptide followed by a 50-amino acid mature peptide (Figure 2B). PA-SUB predicted that the signal peptide of PgD5 directs its product to the extracellular space of plant cells. The peptide parameters obtained from the Expasy-Compute pI/Mw tool showed that PgD5 has a predicted mono-isotopic mass of 5721.56 Da and is highly basic with a net charge of +5 and an isoelectric point above 8.9. Seven different spruce species, namely *P. glauca* and *P. mariana* (native to North America), *P. smithiana*, *P. wilsonii* and *P. orientalis* (native to Asia), and *P. abies* and *P. omorika* (native to Europe) were selected to isolate the corresponding putative defensins from genomic DNA. PCR reactions resulted in amplification of seven genomic copies of *PgD5*, which were sequenced. Alignment analysis of these genomic sequences revealed a very high level of similarity (99%) between the sequences at nucleotide level where the majority of mismatches occurred in the intron region ( Additional file 4). The sequences at the deduced amino acid level were 100% identical, indicating that the peptide is fully conserved within the genus *Picea*.

In the case of *endopiceasin,* primers were designed based on the EST GQ0132.B7_K03 sequence and PCR was carried out to amplify the complete coding sequence of the mature peptide using genomic DNA of *P. glauca*. Genomic amplification was unsuccessful as no product was obtained. These results suggest that the defensin is not encoded by the plant genome itself and we suggest to designate this defensin as *endopiceasin*.

**Figure 3:**
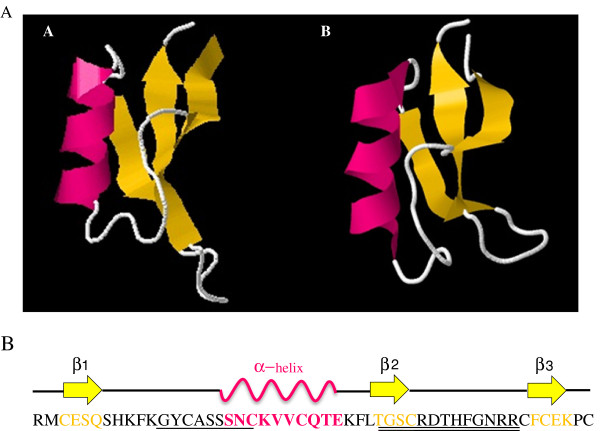
**Protein structure of PgD5.** (**A**) Comparison of the tertiary structure of PgD5 from *Picea glauca* (**A**) and defensin A from *Nicotiana tabacum* (**B**) by homology-based models. The α-helix and β-sheet structures are represented in pink and yellow, respectively. Models were developed using the I-TASSER website for protein structure and function predictions. (**B**) Secondary structure of PgD5. Secondary structure elements (α-helix and β-strands) of PgD5 are given below. Amino acid residues forming the α-core are underlined while the amino acid residues forming the γ-core are double underlined.

Comparative modeling of the deduced amino acid sequence suggested that the tertiary structure of PgD5 closely resembles that of NaD1, a floral defensin from *Nicotiana tabacum* (UniProtKB:P32026). These defensins contain an invariant tetradisulfide array and have the common cysteine-stabilized α/β structure (CSα/β) composed of three antiparallel β-strands and one α-helix which are organized in a βαββ configuration (Figure 3A). Analysis of the PgD5 primary sequence identified γ-core and α-core motifs [52], as shown in Figure 3B.

### Recombinant production and purification of PgD5

**Figure 4:**
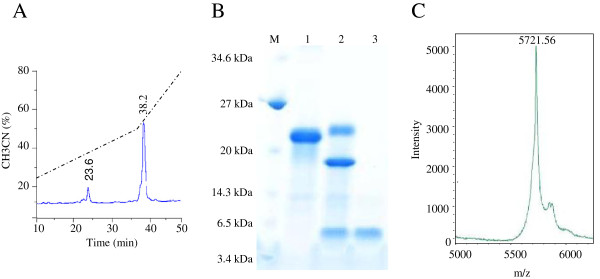
**Purification of recombinant PgD5 produced by *E. coli *BL21 (Origami pLys S) DE3.** (**A**) RP-HPLC chromatography of the His6-SUMO-PgD5 fusion protein after cleavage with the SUMO protease. Elution times are marked. (**B**) SDS-PAGE analysis of the His6-SUMO-PgD5 fusion before and after SUMO protease treatment. Lane M, low molecular weight marker (New England Biolabs). Lane 1, Purified fusion protein by Ni-NTA column. Lane 2, SUMO protease cleavage products after 60 min. Lane 3, Purified PgD5 peptide after SUMO protease digestion and reverse-phase chromatography. (**C**) Mass spectrometric analysis of recombinant PgD5 after separation from the His6-SUMO tag using reverse-phase chromatography.

The His6-SUMO-PgD5 fusion was successfully expressed in *E. coli* BL21 (Origami pLys S) DE3. The recombinant fusion protein of 18 kDa was efficiently produced in soluble form and purified to > 90% purity by Ni-NTA column chromatography in a single step (Figure 4A). The SUMO-tag was removed by the Sumo protease. The resulting protein products were further purified using a C18 reversed phase chromatography column and a TFA/acetonitrile gradient. Recombinant PgD5 eluted at 23.6 min (at 38% solvent B) while the digested SUMO-tag eluted at 38.2 min (at 52% solvent B) (Figure 4B). SDS-PAGE analysis confirmed that PgD5 was successfully separated from the cleaved tag and purified to homogeneity (Figure 4A). The purified peptide was further characterized by MALDI-TOF-MS. MALDI-TOF-MS analysis yielded a mass of 5721.56 Da for the purified peptide, which is 8 Da less than the theoretical mass calculated with the Expasy-Compute pI/Mw tool of 5729.66 Da (Figure 4C). These data indicate that the four disulphide bridges which are common to all plant defensins were correctly formed. Incubation of the peptide in 2 mM DTT reduced the disulphide bridges and completely inactivated the peptide.

### Antimicrobial activity of purified PgD5

**Figure 5:**
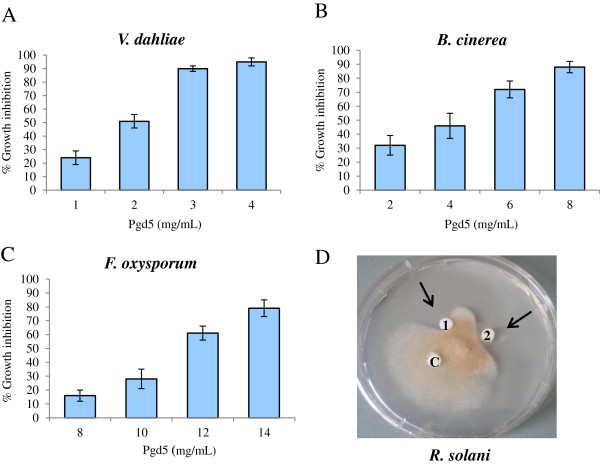
**Recombinant PgD5 possesses antifungal activity.** (**A**-**C**) Dose–response growth inhibition assay. Growth inhibition of *V. dahliae* (**A**), *B. cinerea* (**B**) and *F. oxysporum* (**C**) in the presence of indicated concentrations of recombinant PgD5 was determined by microspectrophotometric readings taken every 24 hours at A_595_ and compared to the untreated fungal controls. The data is represented as the percentage of fungal growth as compared to the untreated control reactions without peptide. The experiment was repeated three times and the standard deviation for each reaction was less than 5%. Growth inhibition was determined after 48 hours of growth for *F. oxysporum* and *B. cinerea* and after 72 hours for *V. dahliae*. (**D**). Effect of purified recombinant PgD5 on mycelial growth of *R. solani*: disk C, sterile distilled water; disks 1 and 2 correspond to 3 and 6 μg of recombinant PgD5, respectively. The arrows indicate zones of growth inhibition.

Antifungal activity of PgD5 was assayed by microspectrophotometry using a dose–response growth inhibition assay. PgD5 significantly inhibited the fungal growth over time in all fungal isolates tested even at very low concentrations. PgD5 was most active against V*. dahliae* (Figure 5A) and *B. cinerea* (Figure 5B), with IC_50_ values of 2 μg/mL and 4 μg/mL, respectively. However, PgD5 was less effective against *F. oxysporum* with an IC_50_ value of 11 μg/mL (Figure 5C). Treatment of *V. dahliae* spores, the causal agent of wilting disease, with a peptide concentration of 3 μg/mL resulted in > 90% growth inhibition, and a concentration of 8 μg/mL completely arrested spore germination (data not shown). The antifungal activity of PgD5 was also determined *in vitro* with a plate assay on *Rhizoctonia solani*. As illustrated in Figure 5D, there are inhibition zones of *R. solani* in areas containing sterile discs of filter paper on which various concentrations of purified PgD5 were applied. Distilled water served as the negative control.

**Figure 6:**
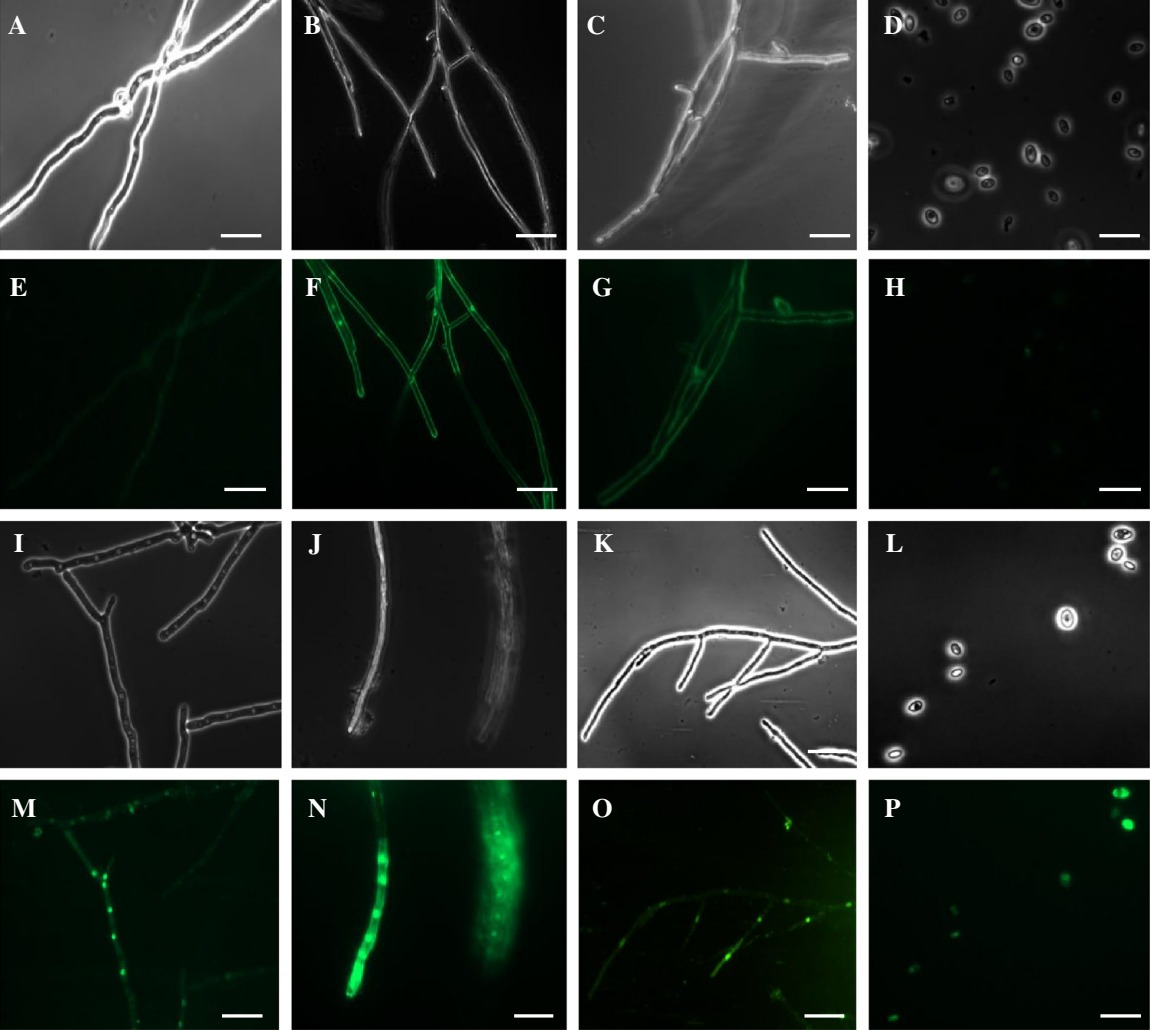
**Fluorescence microscopy analysis of SYTOX Green uptake during the membrane permeabilization assay.** (**A**-**D**). Phase contrast images and (**E**-**H**) fluorescent images of untreated *F. oxysporum, B. cinerea, V. dahliae* and *S. cerevisiae* cells, respectively. (**I**-**L**) Phase contrast images and (**M**-**P**) fluorescent images of PgD5 treated *F. oxysporum, B. cinerea, V. dahliae* and *S. cerevisiae* cells respectively. Fungi were grown for 48h in the presence of PgD5 at peptide concentrations of 11 μg/mL for *F. oxysporum,* 4 μg/mL for *B. cinerea,* and 2 μg/mL for *V. dahliae. S. cerevisiae* cells were grown for 1h in the presence of PgD5 at 11 μg/mL. Afterwards, fungal hyphae and yeast cells were washed with 0.1 M Tris–HCl, pH 8.0, stained with 0.2 mM SYTOX for 30 min at 25°C with periodic agitation and subjected to fluorescent microscopic analysis. Bar = 20 μm.

One characteristic feature of many cationic antimicrobial peptides is their ability to permeabilize the plasma membrane of target organisms. We examined membrane permeabilization using the fluorometric SYTOX Green dye, which is taken up only by cells with compromised plasma membrane. Microscopical analyses of fungal hyphae treated with PgD5 did not reveal increased hyphal branching and morphologically altered hyphae that are typically induced by some plant defensins [7,50,52] (Figure 6). However, PgD5 strongly inhibited elongation of fungal hyphae. By measuring the SYTOX green uptake by fluorescence microscopy we observed that PgD5 induced membrane permeabilization in the three fungi tested. Hyphae of Pgd5-treated *B. cinerea, F. oxysporum* and *V. dahliae* had strong fluorescence in the cytosol (Figure 6M-O), especially in the nuclei, when compared to the untreated fungi that showed no fluorescence (Figure 6E-G). The ability of PgD5 to permeabilize the plasma membrane was also tested in *S. cerevisiae*. By fluorescence microscopy, Pgd5-treated *S. cerevisiae* cells showed strong SYTOX Green fluorescence in the cytosol (Figure 6P) as compared to negative controls (cells grown without peptide) (Figure 6H), indicating that PgD5 also induced membrane permeabilization in the yeast cells. In contrast to these findings, PgD5 was not active against *Candida albicans* at the concentration of 50 μg/mL (data not shown).

### Recombinant PgD5 is heat-stable and moderately sensitive to cations

**Figure 7:**
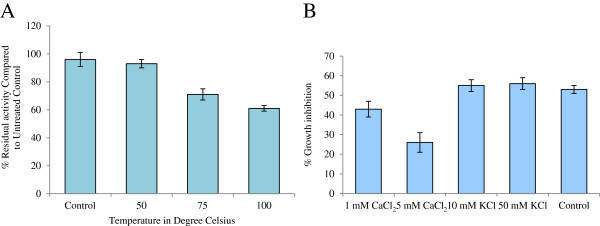
**Thermal stability and effect of cations on PgD5 activity.** (**A**) Temperature stability of PgD5. After heat treatment, the remaining antifungal activity of PgD5 was scored against the control without heat treatment (25°C). (**B**) Effect of the presence of monovalent and divalent cations on PgD5 activity. The antifungal activity of PgD5 (at the dose of 2 μg/mL) was scored against *V. dahliae* by incubating the peptide in the presence of various concentrations of monovalent and divalent cations. The control was treated in the absence of cations in the medium.

PgD5 was tested for its stability at various temperatures using the antifungal growth assay against *V. dahliae*. PgD5 was remarkably stable at temperatures of up to 100°C. Seventy one percent of its antifungal activity was retained after 30 min of treatment at 75°C and 61% at 100°C (Figure 7A). Analysis of the effect of monovalent and divalent cations on antifungal activity of PgD5 showed that the divalent cation Ca^2+^ diminished about 50% the antifungal activity of the peptide at the concentration of 5 mM. The monovalent cation K^+^ had no effect on the antifungal activity of PgD5 at the concentration of 50 mM (Figure 7B).

## Discussion

### Characterization of plant defensin-encoding genes of *Picea glauca*

Plant defensins are a prominent family of cationic peptides that are ubiquitous among the plant kingdom and represent an important part of the plant innate immune system. Previously, only two antifungal proteins have been described in *P. glauca*. The first PgD1 (*Picea glauca* Defensin 1), is a plant defensin of 50 amino acids that displays antifungal activity against *Cylindrocladium floridanum*, *Fusarium oxysporum*, and *Nectria galligena* at 14 μg/mL [43]. The second is an embryo-abundant protein of 109 amino acids that shares 85% similarity with an antifungal protein of *Ginkgo biloba*[44] but lacks the characteristic features of plant defensins.

In the present work, the screening of the *P. glauca* EST database using the TBLASTN algorithm resulted in identification of five new defensin-encoding genes (Figure 1 and Additional file 1). In addition to expanding the list of existing spruce defensins, our research on *P. glauca* defensins and their encoding genes gives insight into conifer biology and genomics. Our results show that the habitual family of defensins found in angiosperms [53] is also present in gymnosperms, giving another example of the high diversity of plant defensins.

Defensins are expressed in different plant tissues including leaves, pods, tubers, fruits, roots, bark and floral organs [2,8,19]. They provide specific defensive capacity to the plant. The cDNA library containing the EST GQ01307_A13 and EST GQ0132.B7_K03 was constructed from mRNA exclusively isolated from actively elongating roots tips free of mycorrhiza, indicating that *PgD5* and *endopiceasin* genes might be expressed in a tissue-specific manner, only in the roots. To the best of our knowledge, these peptides are, along with defensin SPI1 from Norway spruce (*Picea abies*) [54], the only reported plant defensins that are expressed exclusively in the plant root system.

Endopiceasin resembles the structure of classical defensins of fungi, insects and invertebrates with 6 cysteine residues connected by three disulphide-bridges that stabilize the CSαβ motif [2,8,55-57]. PCR for isolation of *endopiceasin* did not result in a product and efforts to amplify the gene by changing the PCR conditions were unsuccessful. DNA isolated from higher plants may originate from symbiotic microbes called endophytes occupying plant tissues at surprisingly high frequencies. Accordingly, few reports have described DNA sequences attributed to gymnosperms and angiosperms, which actually belong to specific endophytes [58,59]. We believe that EST GQ0132.B7_K03 present in *P. glauca* cDNA library originates from an endophyte or epiphytic fungal strain colonizing the root system of *P. glauca*. Experiments are in progress to further identify the origin of the endopiceasin peptide. In this context, the high degree of similarity between endopiceasin and plectasin might be of special interest because plectasin is a potent peptide antibiotic with strong therapeutic potential [49,51]. We also confirmed the similarity of endopiceasin to plectasin by analysing its mode of action. Endopiceasin was only active against gram-positive bacteria and its activity was based on inhibition of cell wall biosynthesis and lipid II binding (manuscript in preparation). In fact, endophytes that colonize internal plant tissues as mutualists have a role in protection of the plant against pathogens [60], and our report indicates that antibacterial defensins from endophytes might play a significant role in such protection.

### Characteristic features of PgD5

PgD5 showed high similarity at amino acid level with related and unrelated plant defensins of the Gymnospermae family (Figure 1B), which supports the idea of a common origin of plant defensins. The presence of a typical secretion signal peptide is one of the characteristic features of plant defensins [61]. Based on the precursor protein structure, PgD5 groups with Class I of the defensin family [2], containing an N-terminal signal peptide followed by the mature defensin domain. This is in contrast to Class II defensin peptides which have an additional C-terminal prodomain.

PgD5 appears completely conserved within the genus *Picea*. There are many reports of plant defensins showing more than 96% identity within the same genus and differing only by one or two amino acids [62-64], which turns into different antimicrobial activities. However, to our best knowledge, this is the first report on a plant defensin being fully conserved within a genus or, at least, within the seven different *Picea* sp. analyzed in this work. This suggests a very specific role for the *PgD5* gene being highly conserved during evolution of the genus *Picea*.

### Antifungal activity of PgD5

PgD5 exhibited strong antifungal activity inhibiting growth of hyphae of four agronomically important pathogens: *B. cinerea,* an airbone pathogen of broadleaved and coniferous trees; *R. solani,* a soilborne pathogen of economically important agricultural plants and *F. oxysporum* and *V. dahliae*, which are the leading causes of wilting disease (Figure 5). Based on the structural and functional similarity, PgD5 was included into the ‘’non-morphogenic” group of plant defensins, which slow down hyphal elongation but do not induce marked morphological distortions [8,18,47]. The activity exerted by PgD5 against the phytopathogenic fungi and *S. cerevisiae* was linked to alteration of fungal membrane permeability. The nuclei of these permeabilized hyphae appeared intact and the cytoplasm unaltered with no signs of granulation of the hyphal cytoplasm, which is observed in some defensins. However, PgD5 was not active against *C. albicans* at concentration of 50 μg/mL. Probably PgD5 interacts with essential plasma membrane structures of *S. cerevisiae* that are not present in *C. albicans*, causing a structural disruption and alteration of the membrane permeability. However, more studies are necessary to discern whether the permeabilization itself is a result of the interaction of the plant defensins with components of the membrane, or with an intracellular target or signaling pathway triggered by the peptide [22].

The antifungal potency of a peptide in the presence of cations is of particular importance for evaluation of defense capacity against microorganisms in plants. Most plant defensins show little or no antifungal activity in the presence of monovalent and divalent cations in the growth medium at concentrations above 50 or 5 mM, respectively [20,64,65]. In these conditions, the interaction of plant defensins with their binding site dictates the specificity of the antifungal activity observed [21,22,66]. In the present study, PgD5 was shown to be highly heat stable and the antifungal activity against *V. dahliae* was unaffected in the presence of monovalent cation K^+^ and moderately affected in the presence of divalent cation Ca^++^ (Figure 7). This makes PgD5 an attractive candidate in the fields of agricultural biotechnology and therapeutic drug design, with possible application as a transferable resistance trait for molecular breeding of crop plants.

## Conclusions

Screening of a EST database of *P. glauca* resulted in the identification of four homologues of *PgD1*, designated *PgD2-5*, and e*ndopiceasin* which is probably of fungal origin. *PgD5* codes for a defensin that is fully conserved within the genus *Picea*. Recombinant PgD5 displayed non-morphogenic antifungal activity, possibly associated with membrane permeabilization, at low concentrations against several fungal plant pathogens. The strong antifungal activity of PgD5 against *V. dahliae* even in high-ionic-strength medium is of special interest, making PgD5 an attractive candidate for engineering pathogen-resistant crops.

## Methods

### Plant material, microbial strains

Leaves from *Picea glauca* were collected from the botanical garden of the University of Oulu, Oulu (Finland). *Escherichia coli* DH5α (maintained in our laboratory) was used for cloning and plasmid amplification. *E. coli* BL21 (origami pLys S) DE3 (Novagen, USA) was used as the expression host. *Candida utilis* and *Saccharomyces cerevisiae* were obtained from our collection in the Pharmazeutische Mikrobiologie des Universitatsklinikums, Bonn, Germany. Yeasts were maintained on potato dextrose agar (PDA). *Rizoctonia solani* (CBS 340.58)*, Fusarium oxysporum* (CBS 619.87), *Botrytis cinerea* (CBS 125.58) and *Verticillium dahliae* (CBS 110272) were purchased from the CBS-KNAW Fungal Biodiversity Center in Utrecht, Netherlands. Fungi were also maintained in PDA at 25°C until sporulation. Spores were harvested in dH_2_O and spore concentration was determined using a Thoma chamber.

### Database searches and primer design

Plant defensin-encoding sequences were discovered by screening the *P. glauca* EST database of the National Centre for Biotechnological Information (NCBI) using TBLASTN. This database was screened using the amino acid sequences of *Picea glauca* defensin 1 [GenBank:AAR84643], *Ginkgo biloba* defensin 1 [GenBank:AY695796.1], *Pinus sylvestris* defensin 1 [GenBank:EF455616.1], *Pinus sylvestris* defensin 2 [GenBank:EF455617.1], *Pinus sylvestris* defensin 3 [GenBank:JN980401.1] and *Pseudoplectania nigrella* defensin [plectasin, Swiss-Prot:Q53I06.1]. Primers were designed based on the EST clones GQ01307_A13 and GQ0132.B7_K03 to identify the complete coding sequences encoded within the ESTs.

### Primer design and defensin genes amplification

Genomic DNA was isolated from *P. glauca* leaves. Leaf tissue was collected, frozen in liquid nitrogen and ground to a fine powder using mortar and pestle. The DNA was extracted by the CTAB method [67].

The genomic DNA isolated from *P. glauca* was used as the template in PCR to isolate the complete coding sequences of *PgD5* and *endopiceasin*. The two primer sets used were forward primer PgD5F (5’-ATGGAGAAGAGAATGGGCAG-3’) and reverse primer PgD5rev (5'-TTAACAGGGTTTCTCGCAGA-3') for *PgD5*, and EST_K03F (5’- ATGAAG TTCACCATCTCCATC-3’) together with reverse primer EST_K03rev (5’-CTAGTAGCACTTGCAAGTGGT-3’) for *endopiceasin*. Amplification conditions were 95°C for 3 minutes, 30 cycles: 95°C for 1 min, 58°C for 45 s, 72°C for 45 s and final extension at 72°C for 7 minutes. The gel-eluted (QIAquick PCR Purification Kit, Qiagen) amplification products were ligated into the pGEM®-T vector (PROMEGA Corporation, Madison, USA) and submitted for sequencing to Eurofins MWG Operon. Nucleotide and deduced amino acid sequence comparisons were made using the BLAST algorithm.

### Sequence analysis of *PgD5* within the genus *Picea*

Leaves from several *Picea* species (*P. abies*, *P. smithiana*, *P*. *mariana*, *P. orientalis, P. omorika* and *P. wilsonii*) were collected from the botanical garden of the University of Bonn, Bonn (Germany) and genomic DNA was isolated as described above. Gene homologues of *PgD5* were isolated from the different *Picea* species as described above. Genomic sequences obtained for the different *Picea* species were analyzed and aligned using ClustalX [68].

### Bioinformatical analysis of the deduced amino acids sequence of *PgD5*

The deduced amino acid sequence of *PgD5* was produced in VectorNTi and analyzed using the BLASTP algorithm. Homologous sequences identified were further aligned using ClustalX [68]. The deduced *PgD5* sequence was also subjected to disulfide bridge analyses using DiANNA [69]; secondary structure analysis as well as homology modeling was done using the I-TASSER server [70,71]. The peptide structure of PgD5 was evaluated for the presence of a signal peptide sequence with SignalP [72] and sub-cellular localization directed by signal peptide was predicted on the Proteome Analyst Specialized Sub-cellular Localization Server (PA-SUB) [73]. The peptide mass prediction was done with the Expasy tool, PEPTIDE-MASS [74].

### Recombinant production of PgD5 in *E. coli*

The Champion™ pET SUMO Expression System was purchased from Invitrogen and used for the recombinant production of PgD5 in *E. coli* BL21 (Origami pLys S) DE3. This system allows for the production of a His6-tag at the N-terminus of the SUMO protein followed by mature PgD5 at the C-terminus (His_6_-SUMO-PgD5). pGEM-T-PgD5 served as the template to prepare the mature PgD5 sequence by PCR. The expected 153-bp fragment encoding the mature form of PgD5 was amplified with the primer set mPgD5FWD (5’-CGGATGTGTGAGTCGCAGA-3’) and mPGD5rev (5’-TTAACAGGGTTTCTCGCAGA-3’). The gel-eluted amplification product was ligated into the linearized pET-SUMO T/A vector and termed pET-SUMO/PgD5. pET-SUMO/PgD5 was transformed into *E. coli* DH5α and the identity and the correct orientation of the sequence was confirmed by sequencing.

The pET-SUMO/PgD5 plasmid that had been constructed was transformed into *E. coli* (Origami pLys S) DE3 expression strain and bacterial colonies were selected by plating onto Luria-Bertani (LB) agar with 25 μg/mL kanamycin, 34 μg/mL chloramphenicol and 12.5 μg/mL tetracycline. A single colony was inoculated to 10-mL LB medium containing antibiotics and grown overnight at 37°C. The overnight cultures were diluted 1:50 (v/v) in 1L of the same medium without antibiotics and grown in the shaking incubator at 37°C and 200 rpm until an optical density (OD_600_) of 0.6. Protein expression was induced by adding 0.4 mM IPTG and subsequent culturing at 25°C overnight with shaking. Cells were harvested by centrifugation at 10,000xg for 15 min at 4°C and the pellets were stored at −80°C until protein extraction.

### Purification of recombinant fusion protein

Each gram of pelleted cells (wet weight) expressing His_6_-SUMO-PgD5 was resuspended in 10 mL BugBuster Protein Extraction Reagent (Novagen) according to the manual, complemented with 1 mM PSMF. The suspensions were gently mixed to facilitate cell lysis and centrifuged at 10,000xg for 20 min at 4°C. The supernatant was applied to the pre-equilibrated Ni-NTA column with binding buffer (50 mM NaH_2_PO_4_, 500 mM NaCl, 20 mM imidazole, pH 7). After extensive washing with binding buffer, the fusion protein was eluted with eluting buffer (50 mM NaH2PO4, 500 mM NaCl, 250 mM imidazole, pH 8). The eluted fractions containing the fusion protein were pooled and dialyzed overnight at 4°C against 10 mM Tris–HCl containing 150 mM NaCl, pH 8, and concentrated to a final 1–5 mL volume. The recombinant PgD5 was digested with 1U SUMO protease per 50 μg fusion protein in the presence of 0.5 mM DTT for 1h at 30°C.

Reverse phase-HPLC was used for the final PgD5 purification step using a reverse-phase C18 column (0.46 cm × 25 cm). The chromatography was performed at a flow rate of 1 mL/min and elution was carried out with a linear gradient from 100% solvent A (TFA 0.1%) to 60% of solvent B (100% acetonitrile containing 0.1 % TFA) over 40 min. The eluate was monitored by on-line measurement of the absorbance at 220 nm. Eluted peptide was freeze-dried, dissolved in distilled water at a final concentration of 100 μg/mL and stored at −20°C.

### Analysis and identification of recombinant PgD5

The purity of eluted PgD5 was evaluated by separating 1 μg peptide on a 15% [w/v] Tris-Tricine gel [75]; after separation the peptide band was visualized by staining with SimplyBlue™ SafeStain (Invitrogen). The molecular mass of purified peptide was analyzed on a MALDI-TOF-MS system (BIFLEX III, Bruker Daltonics GmbH). Aliquots of 1 μL were mixed with 2 μL of α-cyano-4-hydroxycinnamic acid and spotted onto a ground-steel MALDI target plate (Bruker Daltonics GmbH) and air dried at room temperature. Spectra were recorded in the linear mode at a laser frequency of 20 Hz within a mass range of 1000–6000 Da and further analyzed using FlexAnalysis (version 2.0) software (Bruker Daltonics, GmbH). The spectra were externally calibrated using peptide standards. The mass obtained for the peptide was compared to the predicted mono-isotopic mass of the peptide generated with the Expasy-Compute pI/Mw tool.

### Antimicrobial activity assays

The activity of PgD5 against the yeasts *S. cerevisiae* and *C. albicans* was determined using the microbroth dilution method in a 96-well microtiter plate (Nunc U96 microtiter plates) [76]. Briefly, wells were filled with 50 μl of serial dilutions of the peptide and mixed with 50 mL of Potato Dextrose Broth (PDB) containing 2x10^6^ colony forming units (CFU/mL). The 20 mM Tris solution (pH 8.0) was added as the negative control. Experiments were performed at least in three replicates. Microbial growth was assessed by measuring the optical density at 600 nm after 16 h incubation at 30°C without shaking. The minimal inhibitory concentration (MIC) was defined as the lowest concentration of peptide that induced no change in optical density.

Quantitative antifungal activity of PgD5 was assessed using a microspectrophotometric assay in a 96-well microtiter plate (Nunc F96 microtiter plates) [77] with the following fungal pathogens: *F. oxysporum*, *B. cinerea* and *V. dahlia*e. Each well contained 1000 fungal spores in 100 μl half-strength Potato Dextrose Broth (PDB) and purified PgD5 at concentrations ranging from 1 to 50 μg/mL. Control reactions contained no peptide. Plates were incubated in the dark at 23°C for 3 days, with microspectrophotometric readings taken every 24 hours at A_595_. PgD5 defensin activity was scored after 48 h in the case of the fungi *F. oxysporum* and *B. cinerea* and after 72 h in the case of *V. dahliae*, and was expressed as a percentage of growth inhibition. Growth inhibition percentage is defined as 100 × the ratio of the A_595_ of the control minus the A_595_ of the sample over the A_595_ of the control [76]. IC_50_ is defined as the protein concentration at which 50% inhibitions was reached.

Antifungal activity was also tested *in vitro* against the fungus *R. solani*. Fungal discs of uniform size were inoculated at the center of the Petri dish containing about 15 mL PDB and incubated at 25°C. When the mycelia reached 6 cm in diameter, sterile Whatman no.1 filter paper discs (1 cm diameter) were placed on the plate at equal distances from the center. Various quantities of purified PgD5 were added to each disk. The plates were incubated at 25°C and observed periodically until the mycelial growth had enveloped control discs containing sterile distilled water growth inhibition zones had formed around the discs containing active preparations of PgD5.

### Sytox green uptake

The ability of PgD5 to cause plasma membrane permeabilization was measured by SYTOX GREEN (Molecular Probes; Invitrogen Corp, Carlsbad, CA, USA) uptake as described previously [78] on *F. oxysporum, B. cinerea* and *V. dahliae*. The permeabilization assay consisted of 200 mL half-strength PDB containing fungal spores (2 × 10^4^ spores/mL) and PgD5 peptide at concentrations of 11 μg/mL for *F. oxysporum*, 4 μg/mL for *B. cinerea* and 2 μg/mL for the *V. dahliae* isolate. Fungal strains were incubated at 25°C in the presence of PgD5 for 48 hours. Control samples contained no PgD5. After incubation the samples were washed with 0.1M Tris–HCl, pH 8 and stained with 0.2 μM SYTOX Green in 96-well microplates for 30 min at 25°C with periodic agitation, followed by observation in a phase-contrast microscope (Axiophoto Zeiss) equipped with a fluorescence filter set for fluorescein detection (excitation wavelengths, 450 to 490 nm; emission wavelength, 500 nm). Intracellular fluorescence is indicative of a compromised fungal membrane. SYTOX Green uptake in *S. cerevisiae* was measured similarly, except the cell density was approximately 2x10^8^ cells per mL, and incubation was 1h at 30°C in the presence of PgD5 at a concentration of 11 μg/mL.

### Heat stability assessment and effect of ions on purified PgD5 activity

The stability of the purified PgD5 peptide was assessed by an antifungal assay as described above. The heat stability of the peptide was assessed at the final peptide concentration of 2 μg/mL against *V. dahliae* spores, with the peptide being pretreated at 25°C, 50°C, 75°C and 100°C for 30 min, before starting with the antifungal assay. The activity of PgD5 was scored against the control reaction conducted at 25°C. In a similar assay, the effect of the presence of cations on the antifungal activity of PgD5 was also tested. The cations were added to the medium at concentrations of 10 mM and 50 mM for KCl, and 1 mM and 5 mM for CaCl_2_. The effect of monovalent and divalent cations on PgD5 activity was scored against the control reaction conducted without cations.

## Authors' contributions

HGS and HHK supervised the work and helped with conceptual design and manuscript preparation as well as final data analysis. PP, AMP and DR performed conceptual and experimental design and PP was responsible for all the research procedures, data analysis and writing the paper. The authors declare no conflicts of interest. All authors read and approved the final manuscript.

## Supplementary Material

Additional file 1 **Alignment analysis of the deduced amino acid sequence of*endopiceasin*discovered by database searches.** EST GQ0132.B7_K03 (*endopiceasin*) was found by screening the *P. glauca* EST database using the amino acid sequence of plectasin. The percentage similarity compared to plectasin is indicated in the last column. Clich here for file

Additional file 2 **The complete coding sequence and the deduced amino acid sequence of*endopiceasin*encoded within the EST GQ0132.B7_K03.** The underlined amino acids represent the signal peptide, the amino acids in red indicate the pro-peptide while blue amino acids indicate the mature peptide. Clich here for file

Additional file 3 **Alignment of the mature region of endopiceasin with other members of the defensins family.** Alignment analysis of endopiceasin. The percentage similarity compared to endopiceasin is indicated in the last column. [Swiss-Prot:Q53I06.1] plectasin from fungi *Pseudoplectania nigrella*; [GenBank:BAB41027.1] defenisn A from arthropod *Ornithodoros moubata*; [GenBank:ABI52817.1] defensin B from arthropod *Argas monolakensis.* The six-cysteine residues are indicated by yellow and the disulphide bridge pattern is shown below. Clich here for file

Additional file 4 **Alignment analysis of*PgD5*genomic copies isolated from different*Picea*sp.** The differences between the sequences are indicated by yellow. The intron sequences are indicated in lowercase. Clich here for file

## References

[B1] Dixon R Harrison M Lamb C Early events in the activation of plant defense responses Annual Review Phytopath 1994 32 479 501

[B2] Lay FT Anderson MA Defensins-components of the innate immune system in plants Curr Prot Pept Sci 2005 6 1 85 101 10.2174/1389203053027575 15638771

[B3] van Loon LC Rep M Pieterse CMJ Significance of inducible defense-related proteins in infected plants Annu Rev Phytopathol 2006 44 135 162 16602946 10.1146/annurev.phyto.44.070505.143425

[B4] da Cunha L McFall AJ Mackey D Innate immunity in plants: a continuum of layered defenses Microbes Infect 2006 8 5 1372 1381 16697674 10.1016/j.micinf.2005.12.018

[B5] Jones DA Takemoto D Plant innate immunity - direct and indirect recognition of general and specific pathogen-associated molecules Curr Opin Immunol 2004 16 1 48 62 14734110 10.1016/j.coi.2003.11.016

[B6] Broekaert WF Terras FR Cammue BP Osborn RW Plant defensins: novel antimicrobial peptides as components of the host defense system Plant Physiol 1995 108 4 1353 1358 7659744 10.1104/pp.108.4.1353 PMC157512

[B7] Benko-Iseppon AM Galdino SL Calsa TJ Kido EA Tossi A Belarmino LC Crovella S Overview on plant antimicrobial peptides Curr Prot Pept Sci 2010 11 181 188 10.2174/138920310791112075 20088772

[B8] Thomma BP Cammue BP Thevissen K Plant defensins Planta 2002 216 2 193 202 12447532 10.1007/s00425-002-0902-6

[B9] Garcia-Olmedo F Molina A Alamillo JM Rodriguez-Palenzuela P Plant defense peptides Biopolymers 1998 47 6 479 491 10333739 10.1002/(SICI)1097-0282(1998)47:6<479::AID-BIP6>3.0.CO;2-K

[B10] Almeida MS Cabral KM Kurtenbach E Almeida FC Valente AP Solution structure of pisum sativum defensin 1 by high resolution NMR: plant defensins, identical backbone with different mechanisms of action J Mol Biol 2002 315 749 757 11812144 10.1006/jmbi.2001.5252

[B11] Fant F Vranken W Broekaert WF Borremans F Determination of the three dimensional solution structure of raphanus sativus antifungal protein 1 by 1 H NMR J Mol Biol 1998 279 257 270 9636715 10.1006/jmbi.1998.1767

[B12] Shiau YS Horng SB Chen CS Huang PT Lin C Hsueh YC Lou KL Structural analysis of the unique insecticidal activity of novel mungbean defensin VrD1 reveals possibility of homoplasy evolution between plant defensins and scorpion neurotoxins J Mol Recognit 2006 19 441 450 16721719 10.1002/jmr.779

[B13] Kristensen AK Brunstedt J Nielsen JE Mikkelsen JD Roepstorff P Nielsen KK Processing, disulfide pattern, and biological activity of a sugar beet defensin, AX2, expressed in Pichia pastoris Protein Expr Purif 1999 16 3 377 387 10425158 10.1006/prep.1999.1085

[B14] Lay FT Schirra HJ Scanlon MJ Anderson MA Craik DJ The threedimensional solution structure of NaD1, a new floral defensin from nicotiana alata and its application to a homology model of the crop defense protein alfAFP J Mol Biol 2003 325 1 175 188 12473460 10.1016/s0022-2836(02)01103-8

[B15] Yount NY Yeaman MR Multidimensional signatures in antimicrobial peptides Proc Natl Acad Sci 2004 101 19 7363 7368 15118082 10.1073/pnas.0401567101 PMC409924

[B16] Yount NY Andrés MT Fierro JF Yeaman MR The gamma-core motif correlates with antimicrobial activity in cysteine-containing kaliocin-1 originating from transferrins Biochim Biophys Acta 2007 1768 11 2862 2872 17916323 10.1016/j.bbamem.2007.07.024

[B17] Terras FR Eggermont K Kovaleva V Raikhel NV Osborn RW Kester A Rees SB Torrekens S Van Leuven F Vanderleyden J Cammue B Broekaert W Small cysteine-rich antifungal proteins from radish: their role in host defense Plant Cell 1995 7 5 573 588 7780308 10.1105/tpc.7.5.573 PMC160805

[B18] Carvalho A Gomes VM Plant defensins -prospects for the biological functions and biotechnological properties Peptides 2009 30 1007 1020 19428780 10.1016/j.peptides.2009.01.018

[B19] Wong JH Xia L Ng TB A review of defensins of diverse origins Curr Prot Pept Sci 2005 6 85 101

[B20] Osborn RW De Samblanx GW Thevissen K Goderis I Torrekens S Van Leuven F Attenborough S Rees SB Broekaert WF Isolation and characterisation of plant defensins from seeds of asteraceae, fabaceae, Hippocastanaceae and Saxifragaceae FEBS Lett 1995 368 257 262 7628617 10.1016/0014-5793(95)00666-w

[B21] Thevissen K Ferket KK Francois IE Cammue BP Interactions of antifungal plant defensins with fungal membrane components Peptides 2003 24 1705 1712 15019201 10.1016/j.peptides.2003.09.014

[B22] Thevissen K Warnecke DC Francois IE Leipelt M Heinz E Ott C Zahringer U Thomma BP Ferket KK Cammue BP Defensins from insects and plants interact with fungal glucosylceramides J Biol Chem 2004 279 3900 3905 14604982 10.1074/jbc.M311165200

[B23] Thevissen K Cammue BP Lemaire K Winderickx J Dickson RC Lester RL Ferket KK Van Even F Parret AH Broekaert WF A gene encoding a sphingolipid biosynthesis enzyme determines the sensitivity of *Saccharomyces cerevisiae* to an antifungal plant defensin from dahlia (*Dahlia merckii*) Proc Natl Acad Sci 2000 97 17 9531 9536 10931938 10.1073/pnas.160077797 PMC16899

[B24] Thevissen K FrancÂ¸ois IEJA Takemoto JY Ferket KKA Meert EMK Cammue BPA DmAMP1, an antifungal plant defensin from dahlia (*Dahlia merckii*), interacts with sphingolipids from *Saccharomyces cerevisiae* FEMS Microbiol Lett 2003 226 169 173 13129623 10.1016/S0378-1097(03)00590-1

[B25] van der Weerden NL Lay FT Anderson MA The plant defensin, NaD1, enters the cytoplasm of *Fusarium oxysporum* hyphae J Biol Chem 2008 283 14445 14452 18339623 10.1074/jbc.M709867200

[B26] Lobo DS Pereira IB Fragel-Madeira L Medeiros LN Cabral LM Faria J Bellio M Campos RC Linden R Kurtenbach E Antifungal Pisum sativum defensin 1 interacts with *Neurospora crassa* cyclin F related to the cell cycle Biochemistry 2007 46 987 996 17240982 10.1021/bi061441j

[B27] Bloch CJ Richardson M A new family of small (5 kDa) protein inhibitors of insect α-amylases from seeds of sorghum (Sorghum bicolor (L) Moench) have sequence homologies with wheat γ-purothionins FEBS Lett 1991 279 1 101 104 1995329 10.1016/0014-5793(91)80261-z

[B28] Chen KC Lin CY Kuan CC Sung HY Chen CS A novel defensin encoded by a mungbean cDNA exhibits insecticidal activity against bruchid J Agric Food Chem 2002 50 7258 7263 12452641 10.1021/jf020527q

[B29] Liu YJ Cheng CS Lai SM Hsu MP Chen CS Lyu PC Solution structure of the plant defensin VrD1 from mung bean and its possible role in insecticidal activity against bruchids Proteins 2006 63 777 786 16544327 10.1002/prot.20962

[B30] Mendez E Moreno A Colilla F Pelaez F Limas GG Mendez R Soriano F Salinas M de Haro C Primary structure and inhibition of protein synthesis in eukaryotic cell-free system of a novel thionin, gamma-hordothionin, from barley endosperm Eur J Biochem 1990 194 533 539 2176600 10.1111/j.1432-1033.1990.tb15649.x

[B31] Mendez E Rocher A Calero M Girbes T Citores L Soriano F Primary structure of omega-hordothionin, a member of a novel family of thionis from barley endosperm, and its inhibition of protein synthesis in eukaryotic and prokaryotic cell-free systems Eur J Biochem 1996 239 67 73 8706720 10.1111/j.1432-1033.1996.0067u.x

[B32] Melo FR Rigden DJ Franco OL Mello LV Ary MB Grossi de Sa MF, Bloch C Jr: Inhibition of trypsin by cowpea thionin: characterization, molecular modeling, and docking Proteins 2002 48 2 311 319 12112698 10.1002/prot.10142

[B33] Mirouze M Sels J Richard O Czernic P Loubet S Jacquier A François IE Cammue BP Lebrun M Berthomieu P Marquès L A putative novel role for plant defensins: a defensin from the zinc hyper-accumulating plant, *Arabidopsis halleri*, confers zinc tolerance Plant J 2006 47 329 342 16792695 10.1111/j.1365-313X.2006.02788.x

[B34] Spelbrink RG Dilmac N Allen A Smith TJ Shah DM Hockerman GH Differential antifungal and calcium channel-blocking activity among structurally related plant defensins Plant Physiol 2004 135 2055 2067 15299136 10.1104/pp.104.040873 PMC520777

[B35] Kushmerick C Castro MS Cruz JS Bloch C Jr Beirão PS Functional and structural features of g-zeathionins, a new class of sodium channel blockers FEBS Lett 1998 440 302 306 9872391 10.1016/s0014-5793(98)01480-x

[B36] Huang GJ Lai HC Chang YS Sheu MJ Lu TL Huang SS Lin YH Antimicrobial, dehydroascorbate reductase, and monodehydroascorbate reductase activities of defensin from sweet potato [*Ipomoea batatas* (L.) Lam. ‘tainong 57’] storage roots J Agric Food Chem 2008 56 2989 2995 18393437 10.1021/jf072994j

[B37] Chen Z Gallie DR Dehydroascorbate reductase affects leaf growth, development and function Plant Physiol 2006 142 775 787 16891549 10.1104/pp.106.085506 PMC1586046

[B38] Wong JH Zhang XQ Wang HX Ng TB Amitogenic defensin from white cloud beans (*Phaseolus vulgaris*) Peptides 2006 27 2075 2081 16687191 10.1016/j.peptides.2006.03.020

[B39] Ye XY Ng TB Peptides from pinto bean and red bean with sequence homology to cowpea 10-kda protein precursor exhibit antifungal, mitogenic, and HIV-1 reverse transcriptase-inhibitory activities Biochem Biophys Res Commun 2001 285 424 429 11444860 10.1006/bbrc.2001.5194

[B40] Ye XY Ng TB A new antifungal peptide from rice beans J Peptide Res 2002 60 81 87 12102720 10.1034/j.1399-3011.2002.20962.x

[B41] Pavy N Paule C Parsons L Crow JA Morency MJ Cooke JEK Johnson JE Noumen E Guillet-Claude C Butterfield Y Barber S Yang G Liu J Stott J Kirkpatrick R Siddiqui A Holt R Marra M Séguin A Retzel E Bousquet J Mackay J Generation, annotation, analysis and database integration of 16,500 white spruce EST clusters BMC Genomics 2005 6 144 16236172 10.1186/1471-2164-6-144 PMC1277824

[B42] Rigault P Boyle B Lepage P Cooke JE Bousquet J MacKay JJ A white spruce gene catalog for conifer genome analyses Plant Physiol 2011 157 1 14 28 21730200 10.1104/pp.111.179663 PMC3165865

[B43] Pervieux I Bourassa M Laurans F Hamelin RC Séguin A A spruce defensin showing strong antifungal activity and increased transcript accumulation after wounding and jasmonate treatments Physiological and Molecular Plant Pathology 2004 64 331 341

[B44] Sawano Y Miyakawa T Yamazaki H Tanokura M Hatano K Purification, characterization, and molecular gene cloning of an antifungal protein from Ginkgo biloba seeds Biol Chem 2007 388 3 273 280 17338634 10.1515/BC.2007.030

[B45] Zhu S Discovery of six families of fungal defensin-like peptides provides insights into origin and evolution of the CSalphabeta defensins Mol Immunol 2008 45 828 838 17675235 10.1016/j.molimm.2007.06.354

[B46] Kovalyova VA Hut RT Molecular cloning and characterization of Scotch pine defensin 2 Cytol and Genet 2008 42 6 408 412 19253756

[B47] de Beer A Vivier MA Vv-AMP1, a ripening induced peptide from *Vitis vinifera* shows strong antifungal activity BMC Plant Biol 2008 8 75 18611251 10.1186/1471-2229-8-75 PMC2492866

[B48] Fernández-Ocaña A García-López MC Jiménez-Ruiz J Saniger L Macías D Navarro F Oya R Belaj A de la Rosa R Corpas FJ Barroso JB Luque F Identification of a gene involved in the juvenile-to-adult transition (JAT) in cultivated olive trees Tree Genetics & Genomes 2010 6 891 903

[B49] Mygind PH Fischer RL Schnorr KM Hansen MT Sönksen CP Ludvigsen S Raventós D Buskov S Christensen B De Maria L Taboureau O Yaver D Elvig-Jørgensen SG Sørensen MV Christensen BE Kjaerulff S Frimodt-Moller N Lehrer RI Zasloff M Kristensen HH Plectasin is a peptide antibiotic with therapeutic potential from a saprophytic fungus Nature 2005 437 7061 975 980 16222292 10.1038/nature04051

[B50] Wilmes M Cammue BP Sahl HG Thevissen K Antibiotic activities of host defense peptides: more to it than lipid bilayer perturbation Nat Prod Rep 2011 28 8 1350 1358 21617811 10.1039/c1np00022e

[B51] Schneider T Kruse T Wimmer R Wiedemann I Sass V Pag U Jansen A Nielsen AK Mygind PH Raventós DS Neve S Ravn B Bonvin AM De Maria L Andersen AS Gammelgaard LK Sahl HG Kristensen HH Plectasin, a fungal defensin, targets the bacterial cell wall precursor Lipid II Science 2010 328 5982 1168 1172 20508130 10.1126/science.1185723

[B52] Sagaram US Pandurangi R Kaur J Smith TJ Shah DM Structure-activity determinants in antifungal plant defensins MsDef1 and MtDef4 with different modes of action against Fusarium graminearum PLoS One 2011 13 6(4) 10.1371/journal.pone.0018550 PMC3076432 21533249

[B53] Silverstein K Graham M Paape T Van den Bosh K Genome Organization of More 300 Defensin-like Genes In Arabidopsis Plant Physiol 2005 138 4 600 610 15955924 10.1104/pp.105.060079 PMC1150381

[B54] Sharma P Lönneborg A Isolation and characterization of a cDNA encoding a plant defensin-like protein from roots of Norway spruce Plant Mol Biol 1996 31 3 707 712 8790304 10.1007/BF00042244

[B55] Ganz T Defensins: antimicrobial peptides of innate immunity Nature Rev Immunol 2003 3 710 720 12949495 10.1038/nri1180

[B56] Lamberty M Ades S Uttenweiler-Joseph S Brookhart G Bushey D Hoffmann JA Bulet P Insect immunity. Isolation from the lepidopteran *Heliothis virescens* of a novel insect defensin with potent antifungal activity J Biol Chem 1999 274 9320 9326 10092609 10.1074/jbc.274.14.9320

[B57] Lacadena J Martinez del Poxo A, Gasset M, Patino B, Campos-Olivas R, Vazquez C, Martinez-Ruiz A, Mancheno JM, Onaderra M, Gavilanes JG: Characterization of the antifungal protein secreted by the mould Aspergillus giganteus Arc Biochem Biophys 1995 324 273 281 10.1006/abbi.1995.0040 8554319

[B58] Saar DE Polans NO Sørensen PD Duvall MR Angiosperm DNA contamination by endophytic fungi: Detection and methods of avoidance Plant Mol Biol Rep 2001 19 249 260

[B59] Camacho FJ Gernandt DS Liston A Stone JK Klein AS Endophytic fungal DNA, the source of contamination in spruce needle DNA Mol Ecol 1997 6 983 987

[B60] Arnold AE Mejia LC Kyllo D Rojas EI Maynard Z Robbins N Herre EA Fungal endophytes limit pathogen damage in a tropical tree Proc Natl Acad Sci 2003 100 15649 15654 14671327 10.1073/pnas.2533483100 PMC307622

[B61] Broekaert WF Cammue BPA DeBolle MFC Thevissen K DeSamblanx G Osborn RW Antimicrobial peptides from plants Crit Rev Plant Sci 1997 16 3 297 323

[B62] de Beer A Vivier MA Four plant defensins from an indigenous South African Brassicaceae species display divergent activities against two test pathogens despite high sequence similarity in the encoding genes BMC Res Notes 2011 4 1 459 22032337 10.1186/1756-0500-4-459 PMC3213222

[B63] Slavokhotova AA Odintsova TI Rogozhin EA Musolyamov AK Andreev YA Grishin EV Egorov TA Isolation, molecular cloning and antimicrobial activity of novel defensins from common chickweed (Stellaria media L.) seeds Biochimie 2011 93 3 450 456 21056078 10.1016/j.biochi.2010.10.019

[B64] Terras FR Schoofs HM De Bolle MF Van Leuven F Rees SB Vanderleyden J Cammue BP Broekaert WF Analysis of two novel classes of plant antifungal proteins from radish (*Raphanus sativus* L.) seeds J Biol Chem 1992 267 22 15301 15309 1639777

[B65] Terras FR Torrekens S Van Leuven F Osborn RW Vanderleyden J Cammue BP Broekaert WF A new family of basic cysteine-rich plant antifungal proteins from Brassicaceae species FEBS Lett 1993 316 3 233 240 8422949 10.1016/0014-5793(93)81299-f

[B66] Thevissen K Francois IE Aerts AM Cammue BP Fungal sphingolipids as targets for the development of selective antifungal therapeutics Curr Drug Targets 2005 6 8 923 928 16375675 10.2174/138945005774912771

[B67] Pirttilä AM Hirsikorpi M Kämäräinen T Jaakola L Hohtola A DNA Isolation Methods for Medicinal and Aromatic Plants Plant Mol Biol Rep 2001 19 273a

[B68] Thompson JD Gibson TJ Plewniak F Jeanmougin F Higgins DG The ClustalX windows interface: flexible strategies for multiple sequence alignment aided by quality analysis tools Nucl Acids Res 1997 25 4876 4882 9396791 10.1093/nar/25.24.4876 PMC147148

[B69] Ferrè F Clote P DiANNA: a web server for disulfide connectivity prediction Nucl Acids Res 2005 1 33 10.1093/nar/gki412 PMC1160173 15980459

[B70] Ambrish R Kucukural A Zhang Y I-TASSER: a unified platform for automated protein structure and function prediction Nat Protoc 2010 5 725 738 20360767 10.1038/nprot.2010.5 PMC2849174

[B71] Zhang Y I-TASSER server for protein 3D structure prediction BMC Bioinformatics 2008 9 40 18215316 10.1186/1471-2105-9-40 PMC2245901

[B72] Bendtsen JD Nielsen H von Heijne G Brunak S Improved prediction of signal peptides: SignalP 3.0 J Mol Biol 2004 340 783 795 15223320 10.1016/j.jmb.2004.05.028

[B73] Proteome Analyst Specialized Subcellular Localization Server http://www.cs.ualberta.ca/%7Ebioinfo/PA/Sub/index.html

[B74] Expasy proteomics toolshttp://us.expasy.org/tools/

[B75] Schagger H von Jagow G Tricine-sodium dodecyl sulfate-polyacrylamide gel electrophoresis for the separation of proteins in the range from 1 to 100 kDa Anal Biochem 1987 166 368 379 2449095 10.1016/0003-2697(87)90587-2

[B76] Roberts WK Selitrennikoff CP Isolation and characterization of two antifungal proteins from barley Biochim Biophys Acta 1986 880 161 170 3942788 10.1016/0304-4165(86)90076-0

[B77] Broekaert W Terras F Cammue B Vandereyden J An automated quantitive assay for fungal growth inhibition FEMS Microbiol Lett 1990 69 55 60

[B78] Thevissen K Terras FR Broekaert WF Permeabilization of fungal membranes by plant defensins inhibits fungal growth Appl Environ Microbiol 1999 65 5451 5458 10584003 10.1128/aem.65.12.5451-5458.1999 PMC91743

