# Supplementary material for: Identification of defensin-encoding genes of Picea glauca: characterization of PgD5, a conserved spruce defensin with strong antifungal activity
Source: BMC Plant Biol. 2012 Oct 5;12:180. doi: 10.1186/1471-2229-12-180 (PMC3502332; doi:10.1186/1471-2229-12-180)
Supplement: Additional file 1 — Alignment analysis of the deduced amino acid sequence ofendopiceasindiscovered by database searches. EST GQ0132.B7_K03 (endopiceasin) was found by screening the P. glauca EST database using the amino acid sequence of plectasin. The percentage similarity compared to plectasin is indicated in the last column. [file 1471-2229-12-180-S1.pptx]

## Slide 1
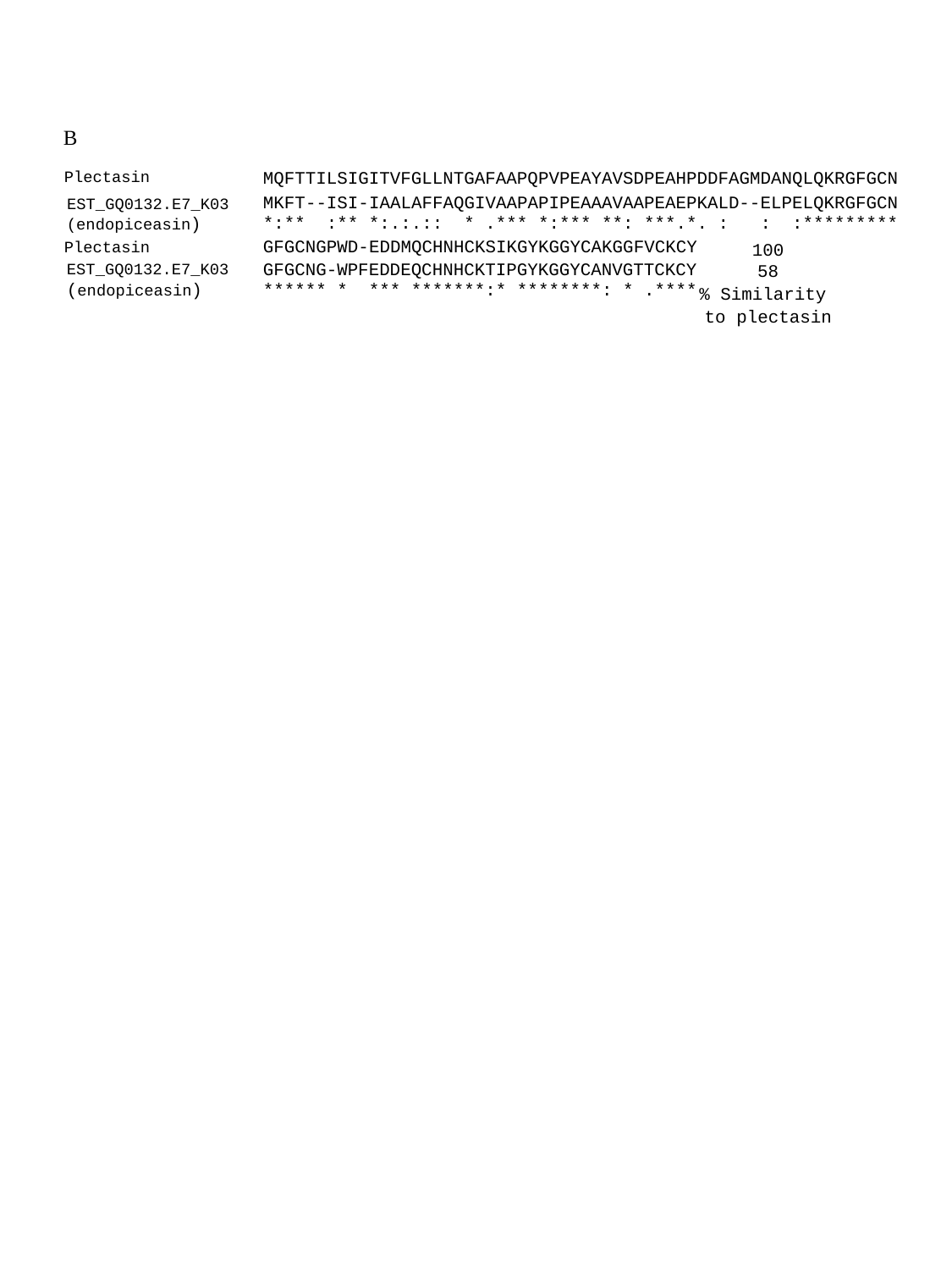

B
Plectasin
MQFTTILSIGITVFGLLNTGAFAAPQPVPEAYAVSDPEAHPDDFAGMDANQLQKRGFGCN
MKFT
--
ISI
-
IAALAFFAQGIVAAPAPIPEA
AAVAAPEAEPKALD
--
ELPELQKRGFGCN
EST_GQ0132.E7_K03
(endopiceasin)
*:** :** *:.:.:: * .*** *:*** **: ***.*. : : :*********
100
58
% Similarity
to plectasin
GFGCNGPWD
-
EDDMQCHNHCKSIKGYKGGYCAKGGFVCKCY
Plectasin
GFGCNG
-
WPFEDDEQCHN
HCKTIPGYKGGYCANVGTTCKCY
EST_GQ0132.E7_K03
(endopiceasin)
****** * *** *******:* ********: * .****
