# Supplementary figures and images for: Identification of defensin-encoding genes of Picea glauca: characterization of PgD5, a conserved spruce defensin with strong antifungal activity
Source: BMC Plant Biol. 2012 Oct 5;12:180. doi: 10.1186/1471-2229-12-180 (PMC3502332; doi:10.1186/1471-2229-12-180)

## Slide 1
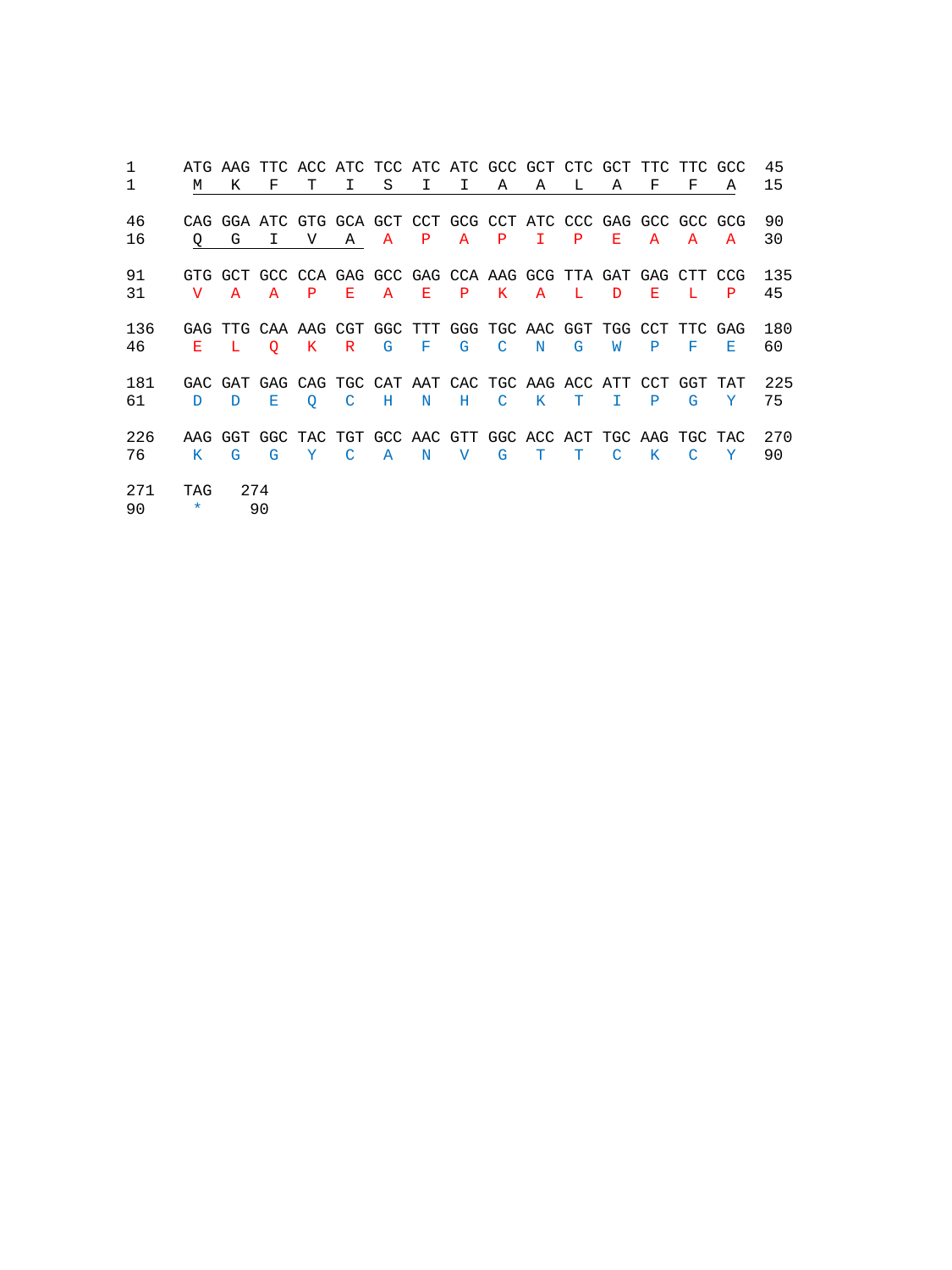

Supplement: Additional file 2 — The complete coding sequence and the deduced amino acid sequence ofendopiceasinencoded within the EST GQ0132.B7_K03. The underlined amino acids represent the signal peptide, the amino acids in red indicate the pro-peptide while blue amino acids indicate the mature peptide. [file 1471-2229-12-180-S2.pptx]

## Slide 1
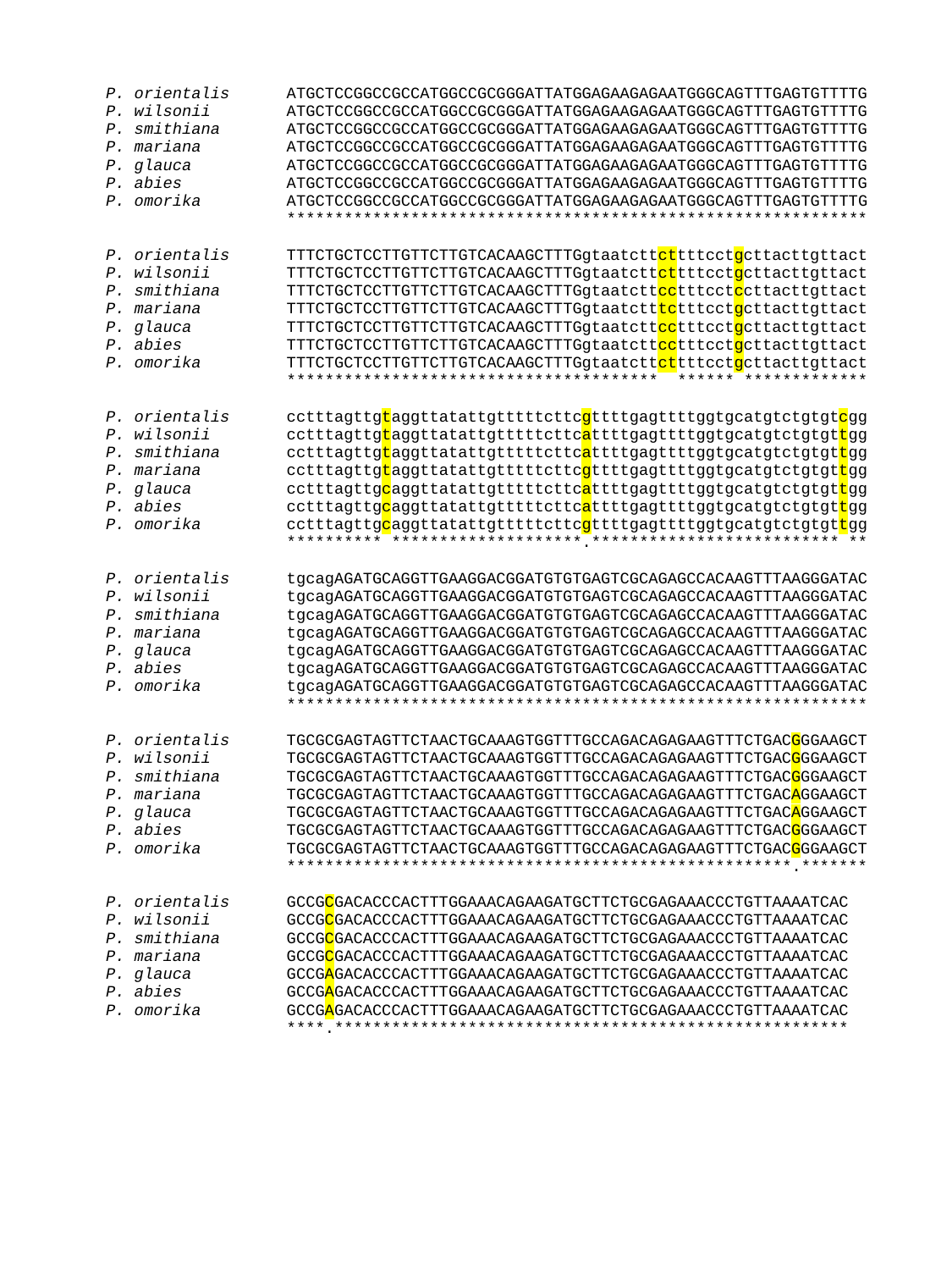

Supplement: Additional file 4 — Alignment analysis ofPgD5genomic copies isolated from differentPiceasp. The differences between the sequences are indicated by yellow. The intron sequences are indicated in lowercase. [file 1471-2229-12-180-S4.pptx]
