# Supplementary material for: Identification of defensin-encoding genes of Picea glauca: characterization of PgD5, a conserved spruce defensin with strong antifungal activity
Source: BMC Plant Biol. 2012 Oct 5;12:180. doi: 10.1186/1471-2229-12-180 (PMC3502332; doi:10.1186/1471-2229-12-180)
Supplement: Additional file 3 — Alignment of the mature region of endopiceasin with other members of the defensins family. Alignment analysis of endopiceasin. The percentage similarity compared to endopiceasin is indicated in the last column. [Swiss-Prot:Q53I06.1] plectasin from fungi Pseudoplectania nigrella; [GenBank:BAB41027.1] defenisn A from arthropod Ornithodoros moubata; [GenBank:ABI52817.1] defensin B from arthropod Argas monolakensis. The six-cysteine residues are indicated by yellow and the disulphide bridge pattern is shown below. [file 1471-2229-12-180-S3.pptx]

## Slide 1
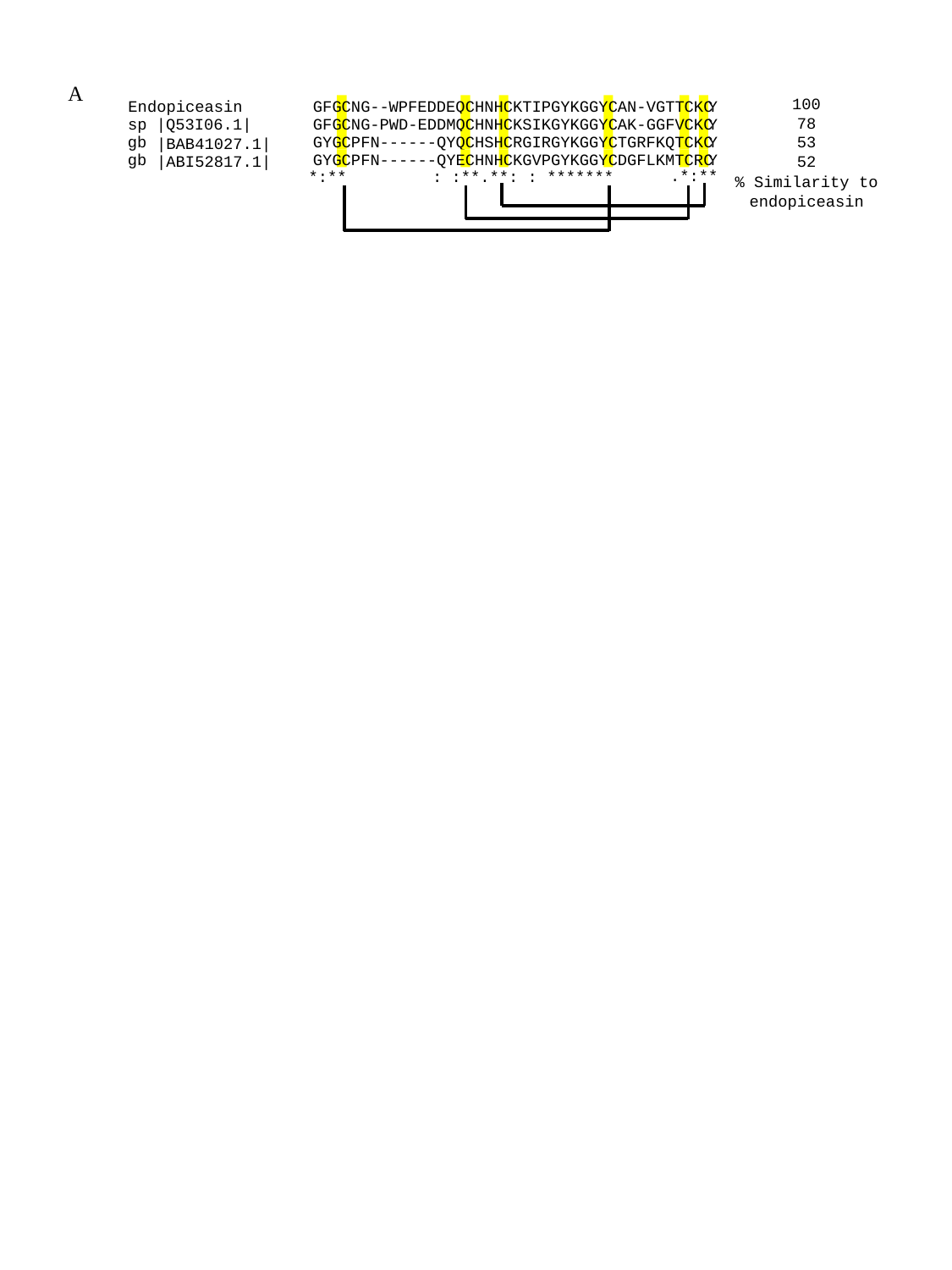

A
100
78
53
52
% Similarity to endopiceasin
Endopiceasin
GFG
C
N
G
--
WPFEDDEQ
C
HNH
C
KTIPGYKGGY
C
AN
-
VGTT
C
K
C
Y
sp |Q53I06.1|
GFG
C
N
G
-
PWD
-
EDDMQ
C
HNH
C
KSIKGYKGGY
C
AK
-
GG
FV
C
K
C
Y
gb
GYG
C
P
FN
------
QYQ
C
HSH
C
RGIRGYKGGY
C
TGRFKQT
C
K
C
Y
|BAB41027.1|
gb
GYG
C
P
FN
------
QYE
C
HNH
C
KGVPGYKGGY
C
DGFLKMT
C
R
C
Y
|ABI52817.1|
.*:**
*:**
: :**.**: : *******
